# A Study to Evaluate the Association Between Thyroid Function and Serum Potassium Level in Diagnosed Cases of Diabetic Nephropathy

**DOI:** 10.7759/cureus.18569

**Published:** 2021-10-07

**Authors:** Polina Boruah, Arup Baruah, Bhupen Barman, Chandan Nath, Ranendra Hajong, Narang Naku

**Affiliations:** 1 Department of Biochemistry, North Eastern Indira Gandhi Regional Institute of Health and Medical Sciences, Shillong, IND; 2 Department of General Surgery, North Eastern Indira Gandhi Regional Institute of Health and Medical Sciences, Shillong, IND; 3 Department of Internal Medicine, North Eastern Indira Gandhi Regional Institute of Health and Medical Sciences, Shillong, IND; 4 Department of Surgery, North Eastern Indira Gandhi Regional Institute of Health and Medical Sciences, Shillong, IND

**Keywords:** thyroid stimulating hormone (tsh), diabetes mellitus, serum potassium, diabetic nephropathy (dn), tri-iodothyronine

## Abstract

Introduction

Coexistence of diabetes mellitus and thyroid diseases is common. One of the main microvascular complications of diabetes is diabetic nephropathy (DN) and it is found to be the leading cause of chronic kidney disease. The aim of the present study was to assess the association between hypothyroidism and serum potassium levels in diabetic nephropathy patients.

Materials and methods

A cross-sectional study was conducted from March 2020 to January 2021. We enrolled 100 patients with DN along with 50 healthy controls belonging to the same localities. Serum potassium, creatinine, thyroid-stimulating hormone (TSH) and total triiodothyronine (T3) levels of all the cases were measured to establish the correlation of serum potassium along with each parameter separately.

Results

Serum potassium, creatinine, TSH levels were increased in all the cases of diabetic nephropathy showing positive correlations of serum potassium with serum TSH and serum creatinine levels with correlation coefficient values 0.71 and 0.7 respectively and serum T3 levels were decreased in all the cases significantly showing negative correlation with serum potassium levels with correlation coefficient value -0.34.

Conclusion

Estimation of serum TSH and T3 levels along with serum potassium levels is important and helpful in patients with diabetic renal disease. Changes in thyroid parameters like decreased TSH or increased T3 are significantly associated with deterioration in the severity of renal function in diabetic patients.

## Introduction

Thyroid diseases and diabetes mellitus (DM) are the two most common and closely related endocrine disorders seen in clinical practice. Diabetes and thyroid disorders are mutually influenced by each other and there are various studies about associations between both conditions conducted so far. Thyroid hormones play a pivotal role in the regulation of renal hemodynamics, glomerular filtration rate (GFR) and water and electrolyte homeostasis [1]. Hyperthyroidism is accompanied by increased renal blood flow and GFR. Low thyroid function is associated with renal impairment via reducing renal plasma flow (RPF) or altering perfusion and low GFR as well as alteration in the renin-angiotensin system [2,3]. In addition, it is also associated with a decrease in sodium reabsorption, decrease in renal potassium excretion, and decreased renal ability to dilute urine, resulting in hyperkalemia and hyponatremia.

The effects of thyroid hormone on electrolytes and minerals are complex and the basic mechanisms are not very cleared. Thus, the present study was conducted to assess the effect of different thyroid hormones in the variation of serum potassium levels in diabetic nephropathy patients with low thyroid status. The aim of the study was to assess if there is any association between hypothyroidism and serum level of potassium in diabetic nephropathy patients.

## Materials and methods

A hospital-based cross-sectional study was conducted in a tertiary care hospital at Shillong, Meghalaya in Northeast India, and the patients were divided into two groups: (i) a case group (diabetic nephropathy patients, as per American Diabetic Association criteria), and (ii) a control group (healthy individuals from the same ethnic groups). The sample size is calculated by using the following formula:

n = N*X / (X + N - 1),

where,

X = Zα/22 ­*p*(1-p) / MOE2,

and Zα/2 is the critical value of the Normal distribution at α/2 (e.g., for a confidence level of 95%, α is 0.05 and the critical value is 1.96), MOE is the margin of error, p is the sample proportion, and N is the population size.

Based on the above sample size estimations, 100 patients with DN were included for the case group between March 2020 and January 2021 from the biochemistry department. The exclusion criteria were: the patients who had undergone surgical intervention, chemotherapy, or other drugs that can alter the thyroid parameters like amiodarone, propranolol, and the patients with complications of other diseases such as cardiovascular and respiratory disorders, and pregnant ladies. Women were not on any hormonal replacement therapy at the time of the study. Serum thyroid-stimulating hormone (TSH) and total triiodothyronine (T3) ­were measured by chemiluminescent method, serum creatinine and potassium were estimated by photometric creatinase and ion-selective electrode (ISE) methods, respectively. Biological reference ranges for serum potassium: 3.5-5.0 mmol per liter, serum creatinine for female: 0.5-1.1 mg/dl, male: 0.6-1.2 mg/dl, TSH: 0.45-4.5 μIU/ml, total T3: 0.87-1.78 ng/ml. The parameters of this study were estimated in the instruments UniCel DxI 600 Access Immunoassay System and AU5600 system of Beckman Coulter (Beckman Coulter Inc., Brea, CA, USA) in the clinical biochemistry department of the institute. For comparison, 50 healthy individuals from similar socio-economic backgrounds and from the same ethnicity and geographical areas were also enrolled in the control group for the study during the same period.

Statistical analysis

All data were included and analysed in Microsoft Office Excel 2010 (Microsoft® Corp., Redmond, WA, USA). Association between different thyroid parameters (TSH and T3) and serum potassium were calculated by correlation coefficient.

Ethical approval was obtained from our Institutional Review Board vide letter No. NEIGR/IEC/2013/29. Written consent was taken from all individuals in the study.

## Results

Serum of all the cases was collected and analysed for potassium, creatinine, TSH and total T3 levels to establish the correlation of serum potassium along with serum TSH, serum creatinine and serum total T3 levels separately. Serum potassium, creatinine, TSH levels were increased and serum Total T3 levels were decreased significantly in all the cases of diabetic nephropathy.

**Figure 1:**
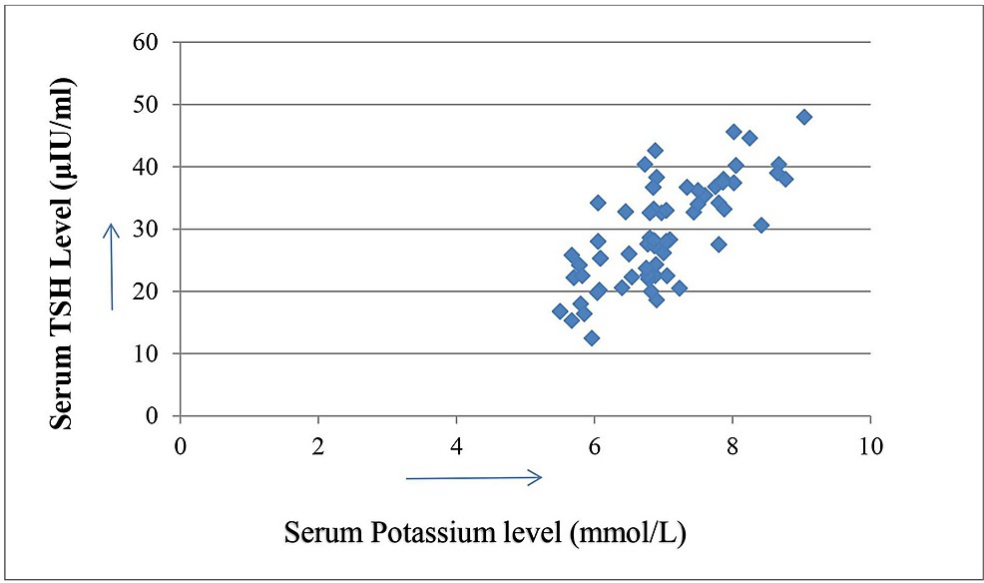
Correlation of serum potassium with thyroid-stimulating hormone (TSH) levels in diabetic nephropathy Correlation coefficient = 0.71

**Figure 2:**
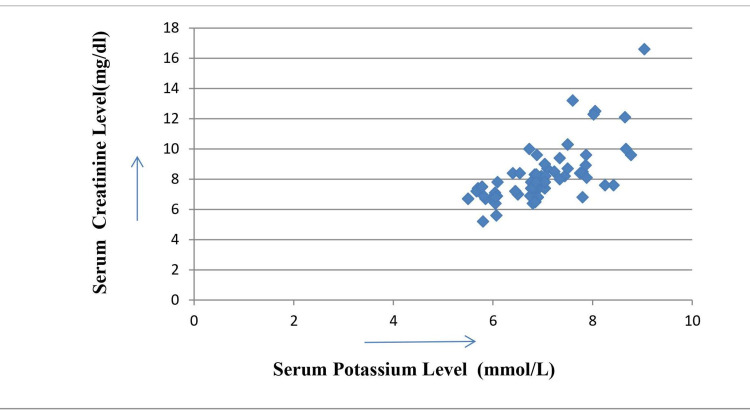
Correlation of serum potassium level with serum creatinine levels in diabetic nephropathy Correlation coefficient = 0.7

**Figure 3:**
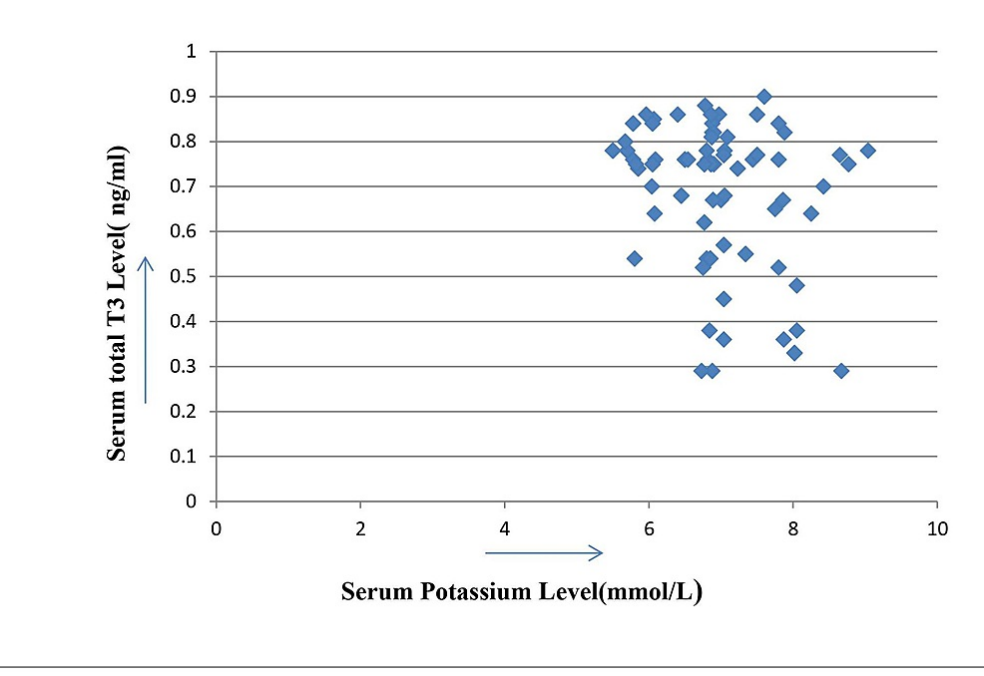
Correlation of serum potassium level with serum T3 levels in diabetic nephropathy Correlation coefficient = -0.34

Serum potassium levels were showing positive correlations with serum TSH and serum creatinine levels with correlation coefficient values 0.71 and 0.7, respectively (Figures 1, 2) but in the same group serum potassium levels were showing negative correlation with serum total T3 levels with correlation coefficient value -0.34 (Figure 3). The calculated r-values of 0.71 and 0.7 are highly significant with two-tailed distributions. The r-value of -0.34 is significant at 5% level (p < 0.05).

## Discussion

In the present study, we evaluated the associations between different thyroid parameters and serum potassium level in patients with DN. Our results showed marked elevation of serum TSH, potassium levels and a significant decrease of serum total T3 levels in all the cases of DN. This was maybe due to reduced renal perfusion in presence of hypothyroidism which leads to decreased clearance of serum creatinine. Thus hypothyroidism causes further deterioration of renal functions as evident by increased serum creatinine level of these cases aggravating renal tubular acidosis which leads to increased serum potassium levels (hyperkalemia). Several studies have reported a close interrelationship between end-stage renal disease and hypothyroidism [4-6]. In the largest data by Lo et al., the third National Health and Nutrition Examination Survey, a nationally representative 14,623 samples of the United States showed a higher prevalence of hypothyroidism with lower levels of eGFR categories, occurring in 5.4%, 10.9%, 21.0%, 23% and 23.1% patients with stage 1-5 chronic kidney disease (CKD), respectively [7]. Thyroid hormones play an important role in the regulation of normal cell function and differentiation by interacting with intracellular thyroid hormone receptors and transcriptional co-regulatory factors (coactivators and corepressors) [8-9].

The sodium pump (Na+-K+-ATPase) is a membrane-bound enzyme that helps to maintain the Na+ and K+ gradients across the plasma membrane of various types of tissues [10]. Thyroid hormones like TSH and T3 induce Na+-K+-ATPase activity indifferent responsive tissues and thus regulate various types of Na+-K+-ATPase isoforms. One isoform such as T3 response elements (TRE) exists in the specific 5 flanking region of α- and β subunits of Na+-K+-ATPase [11,12]. T3 helps in the induction of the activity of Na+-K+-ATPase by increasing the synthesis of Na+-K+-ATPase mRNA or protein [13-16]. As well as, T3 is also responsible for the gene expression of Na+-K+-ATPase in various tissues by some tissues type-specific manners viz. T3 upregulates Na+-K+-ATPase activity in some organs or tissues like skeletal muscle, kidney and liver of rats, however, it inhibits synaptosomal Na+-K+-ATPase activity in the cerebral cortex of rats [17-18]. Moreover, several studies from across the globe have been reported that an increased serum potassium level in hypothyroidism and decreased level in hyperthyroidism are due to increased Na+-K+-ATPase activity [19-21].

Thyroid function can influence renal circulation, e.g., low thyroid hormone is known to be associated with decreased renal plasma flow and low glomerular filtration rate (GFR). Thyroid hormone also regulates GFR by affecting renal blood flow through afferent and efferent arterioles and also the vascular resistance in adult kidneys. Data from several epidemiological studies have shown associations of estimated renal function with thyroid function, including an inverse association between estimated GFR (eGFR) and serum TSH levels, as well as the risk of developing subclinical and overt hypothyroidism across all the spectrum of chronic kidney disease (CKD) [22-24]. Conversely, hypothyroidism and subclinical hypothyroidism have been found to lead to a higher prevalence of CKD over time [25].

## Conclusions

It is found in our present study that an increase in the level of serum TSH along with a decrease in serum total T3 level is associated with deterioration in the severity of renal function in diabetic patients as evident with an increase in serum creatinine and serum potassium levels. Hence to arrest or minimise the harmful impact of high TSH and low T3 levels appropriate doses of thyroid hormone supplementation can be recommended if thyroid function is routinely estimated in these patients with DN.
